# Vacuum suction dressing for infected open calcaneal fractures: a case series

**DOI:** 10.1093/jscr/rjaf175

**Published:** 2025-04-15

**Authors:** Obaid Ur Rehman, Ameer Hamza Mahmood Ul Hassan, Nehala Nooz, Muhammad Hassan, Imtiaz Ahmed Shakir, Abdur Rehman, Javed Iqbal

**Affiliations:** Trauma and Orthopedics Department, Benazir Bhutto Hospital, Chandni Chowk, Murree Road, Rawalpindi 23000, Pakistan; Trauma and Orthopedics Department, Benazir Bhutto Hospital, Chandni Chowk, Murree Road, Rawalpindi 23000, Pakistan; Trauma and Orthopedics Department, Benazir Bhutto Hospital, Chandni Chowk, Murree Road, Rawalpindi 23000, Pakistan; Trauma and Orthopedics Department, Benazir Bhutto Hospital, Chandni Chowk, Murree Road, Rawalpindi 23000, Pakistan; Trauma and Orthopedics Department, Benazir Bhutto Hospital, Chandni Chowk, Murree Road, Rawalpindi 23000, Pakistan; Department of ENT and Head and Neck Surgery, Rawalpindi Medical University, Tipu Road, Chamanzar Colony, Rawalpindi 46000, Pakistan; Nursing Department, Centre for Communicable Disease, Hamad Medical Corporation, Rayan Road, Doha 3050, Qatar

**Keywords:** closure, vacuum assisted, negative-pressure wound therapy, topical negative pressure therapy, vacuum assisted closure, fractures, bone, fractures, open

## Abstract

This study reports on the management of open calcaneal fractures using vacuum suction dressing (VSD) in three cases at a tertiary care hospital. The cases include a 40-year-old man with bilateral calcaneal fractures, a 5-year-old girl with an avulsed Achilles tendon, and a 16-year-old girl with an infected open foot fracture. VSD was utilized in each case to promote wound healing, prevent severe complications like infection and amputation, and reduce the need for invasive surgery. All patients showed significant improvement, with successful wound healing and recovery over the follow-up period. The findings suggest that VSD is an effective non-surgical option for managing complex open calcaneal fractures, particularly in cases with high infection risks. However, further research is needed to establish standardized protocols for its long-term efficacy across diverse populations. The study highlights the need for more data on VSD in fracture management to validate its benefits.

## Introduction

Open ankle fractures are rare but carry a high risk of wound complications and infection [1]. These injuries, often caused by high-energy trauma, require specialized management strategies, especially in open calcaneal fractures, which are linked to significant complications [2]. Vacuum suction dressings (VSD) have emerged as a promising adjunct for promoting wound healing and functional recovery [3].

The management of calcaneal fractures has evolved over the past decade, with open treatment gaining popularity despite its association with complications such as wound necrosis, compartment syndrome, chronic pain, and tendon issues [4].

VSD, introduced over the past two decades, offers significant advantages in managing soft tissue defects by improving wound healing and infection control. However, its role in treating calcaneal fractures remains underexplored [5].

High-energy open calcaneal fractures often result in high infection rates, uncertain outcomes, and a risk of amputation. This study highlights the potential of VSD to mitigate these complications [6]. The case series adheres to the Preferred Reporting Of CasE Series in Surgery (PROCESS) criteria [7].

## Case reports

### Case 1

**Figure 1:**
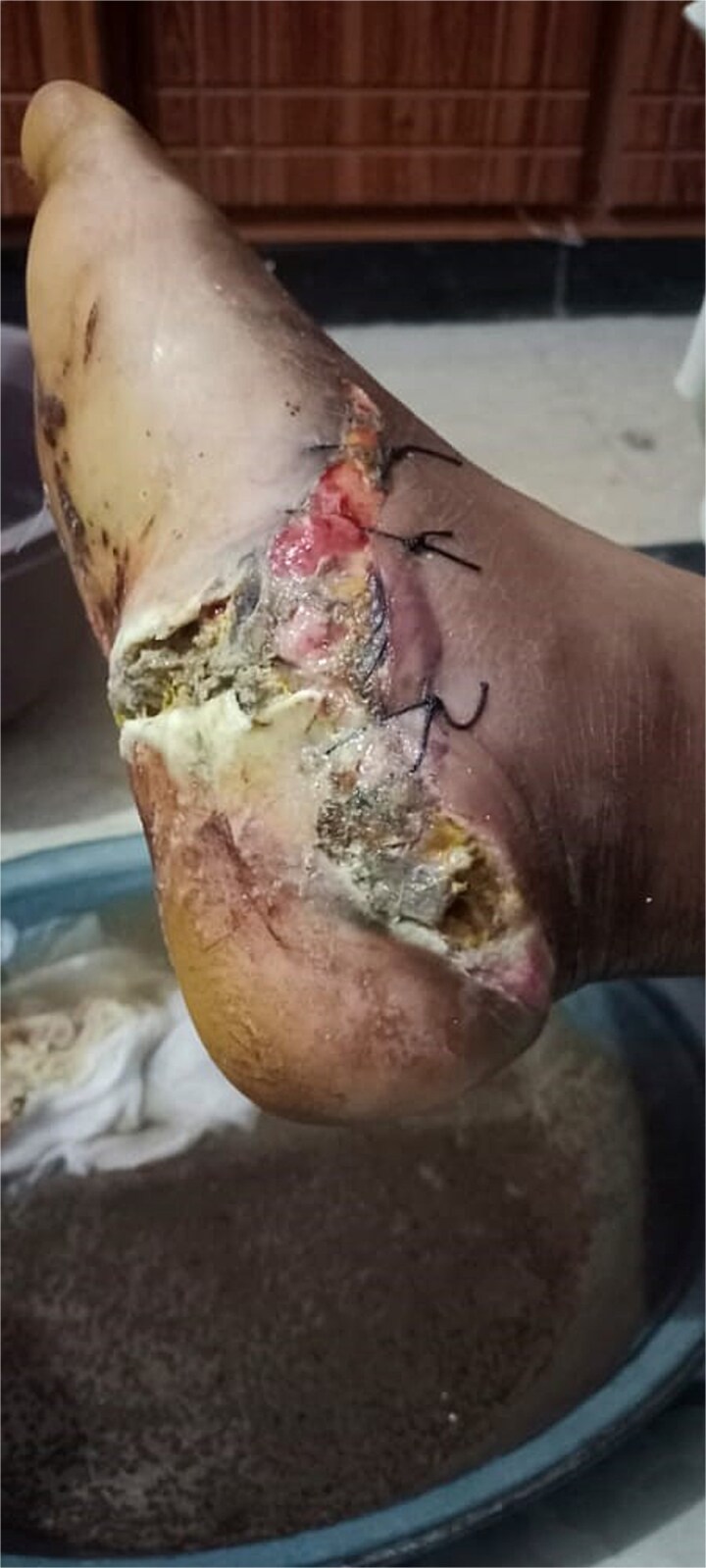
Initial presentation of bilateral open calcaneal fractures in Case 1, showing extensive tissue damage on the right foot.

**Figure 2:**
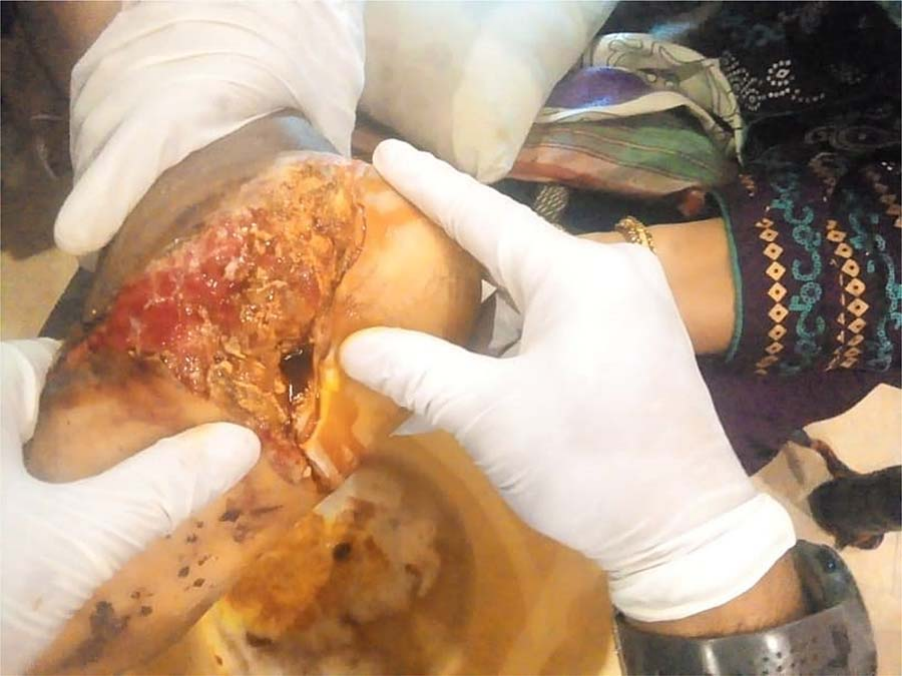
Initial presentation of bilateral open calcaneal fractures in Case 1, showing tissue damage on the left foot.

A 40-year-old obese South-East Asian male fell 15 feet, sustaining bilateral open calcaneal fractures. Initial management involved loose sutures and dressings at another facility, but wound infection and necrosis developed, prompting a recommendation for below-knee amputation. Orthopedic evaluation classified the fractures as Type 3B on the right foot and Type 2 on the left foot. Figures 1 and 2 show the initial state of the wounds.

**Figure 3:**
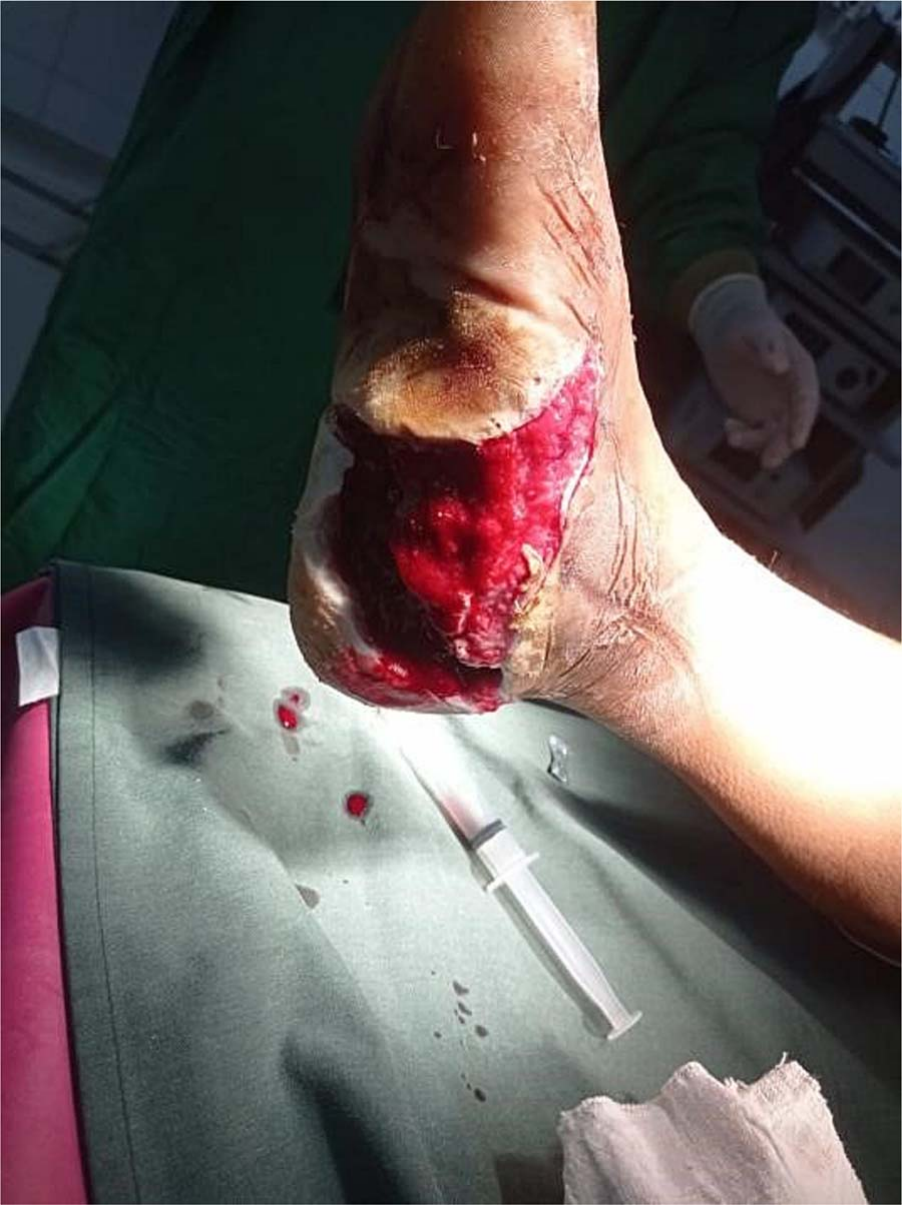
Granulation tissue development on Day 3 of VSD treatment in Case 1.

**Figure 4:**
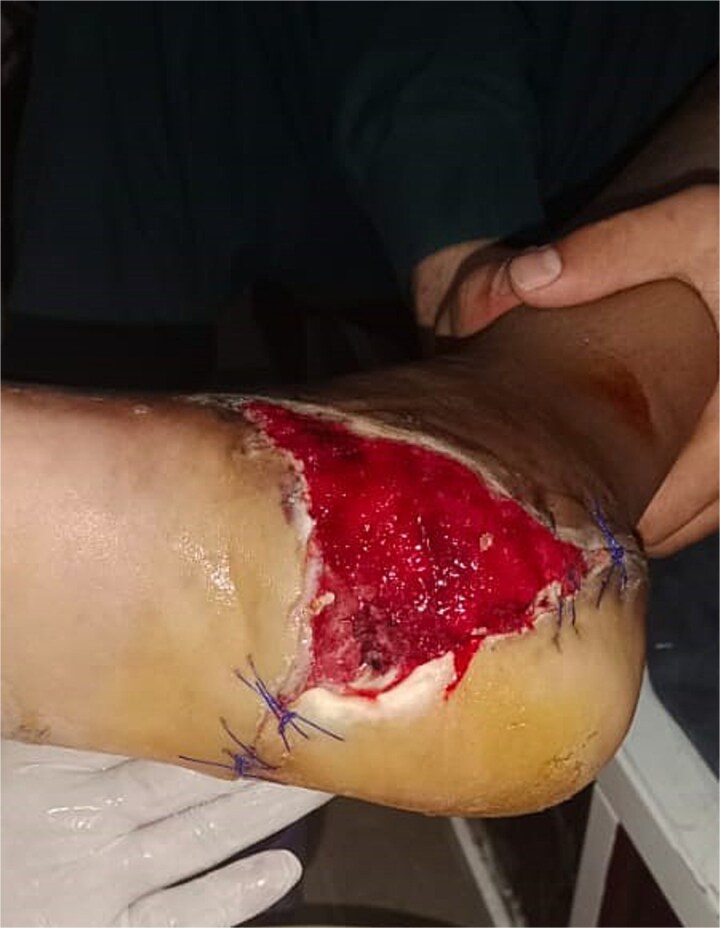
Wound healing progress in Case 1 after 1 week of follow-up.

**Figure 5:**
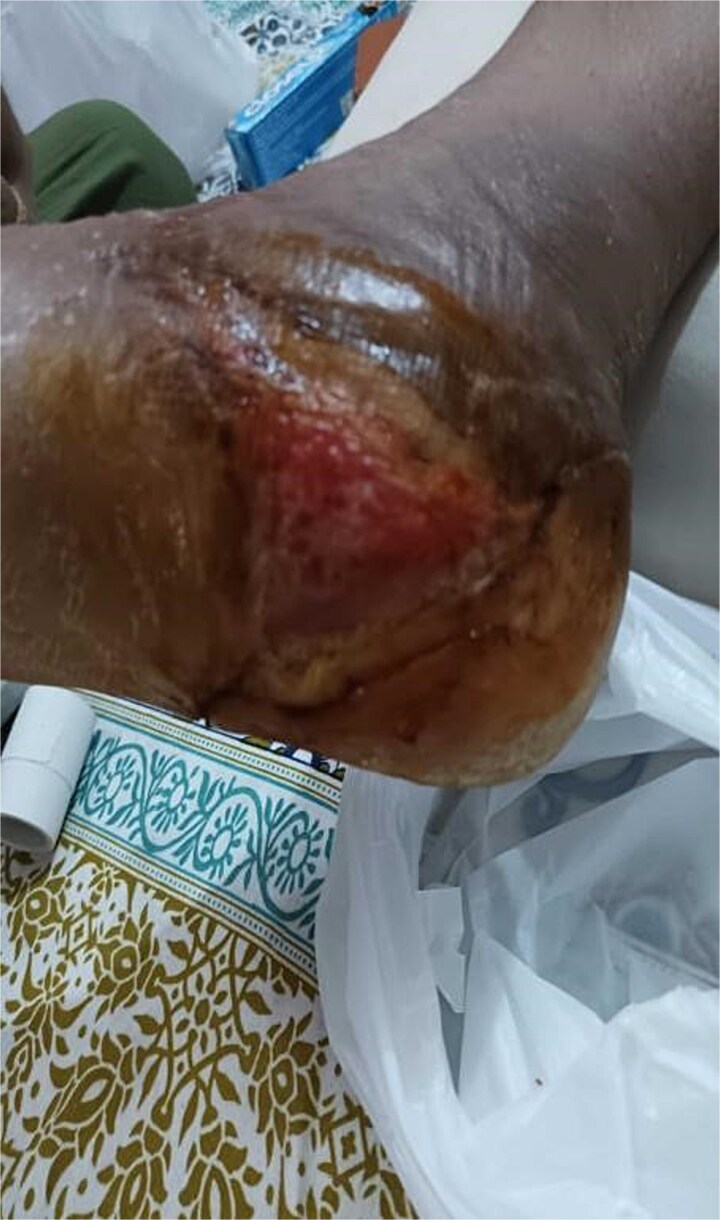
Further improvement in wound healing in Case 1 after 2 months of follow-up.

**Figure 6:**
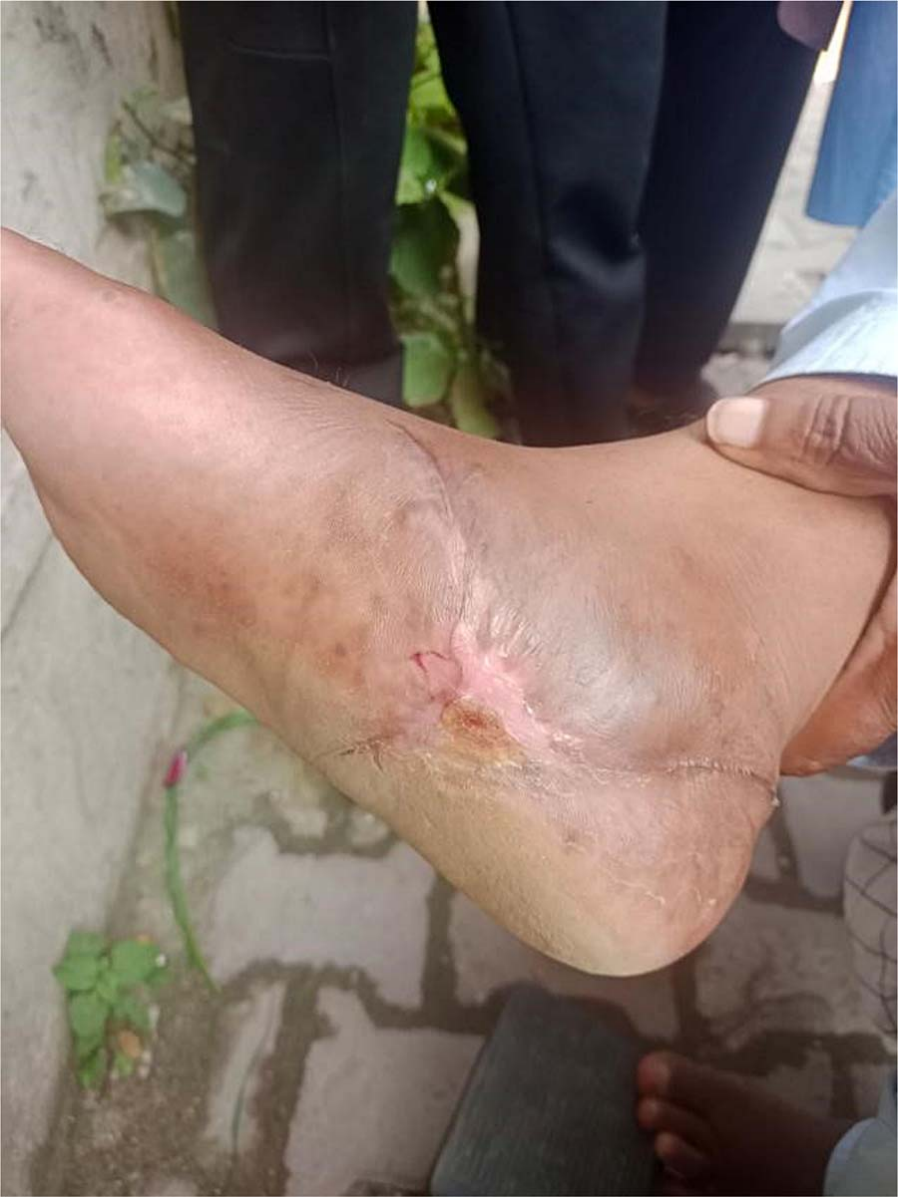
Complete wound closure in Case 1 at the end of the 4-month follow-up.

Surgical debridement under general anesthesia removed necrotic tissue and shattered bone fragments, followed by VSD application. After the first dressing change on Day 3, granulation tissue was observed (Fig. 3). The wound healed completely over 4 months, as shown in Figs 4–6, with concurrent physiotherapy enabling a return to weight-bearing activities.

### Case 2

A 5-year-old South-East Asian girl sustained a posterior foot and leg injury from motorcycle wheel spikes, leading to an avulsed Achilles tendon with calcaneal bone fragments and a 5 × 7 cm open wound. Initial emergency management included tendon repair and back-slab application, but this resulted in wound infection with pus and odor. Upon reassessment, surgical debridement under aseptic conditions removed necrotic tissue and bone, and VSD was initiated.

**Figure 7:**
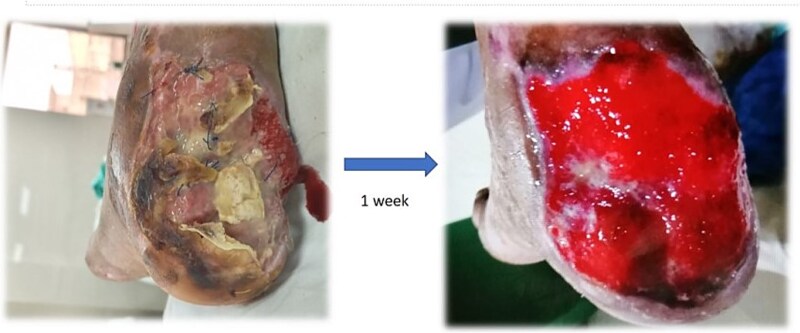
Healthy granulation tissue development in Case 2 after 1 week of VSD treatment.

After 1 week of VSD treatment, healthy granulation tissue appeared without signs of infection (Fig. 7). The patient was referred to a plastic surgeon for wound closure.

### Case 3

**Figure 8:**
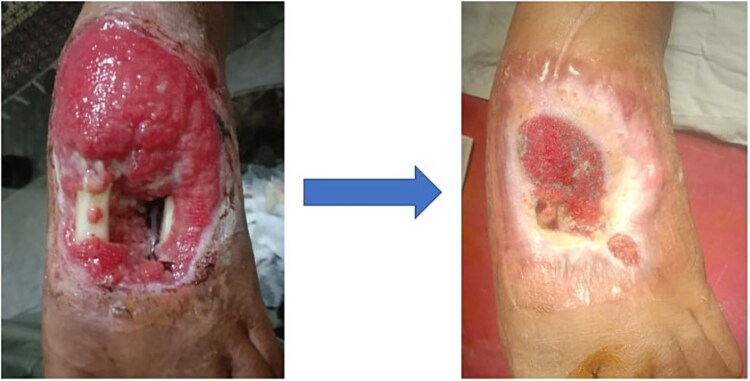
Initial presentation and follow up of an infected open foot fracture with bone loss in Case 3.

A 16-year-old South-East Asian female suffered an open left foot fracture after a car ran over her foot, resulting in an infected wound and bone loss of the second metatarsal. Previous consultations with multiple specialists delayed definitive treatment. Figure 8 illustrates the initial wound condition.

Surgical debridement was followed by VSD application, which resulted in granulation tissue formation by Day 20. The wound was fully closed by Day 37, with no recurrence of infection. The patient resumed normal activities without further complications.

## Discussion

Open calcaneal fractures are high-energy, severe injuries that carry a significant risk of patient morbidity due to the high incidence of concurrent orthopedic and whole-body system injuries. Wound complications due to decreased blood supply and weight bearing of the heel pose problems to different sorts of grafts. Amputation is more likely to be necessary after Type III open injuries. In addition to immediate debridement and irrigation, tetanus prophylaxis and intravenous antibiotics should be used to treat these injuries [8].

In contrast to closed calcaneal fractures, there is comparatively little literature on the surgical management of open fractures. The few studies available use non-standardized treatment protocols, have small patient numbers, and have inconsistent rates of infection and complications [9]. A systematic review concluded that open calcaneal fractures have a comparatively high risk of complications, varying depending on the injury’s extent [2].

As in our case, the calcaneum was grossly comminuted; in line with other investigations, final fracture stabilization was most likely only required during the first irrigation and debridement procedure once all of the soft tissue had been covered [10]. However, other research suggests that VSD-assisted definitive fracture stabilization may be more effective [3].

Under VSD, a wound sealed with a specially made dressing is subjected to sub-atmospheric pressure. A tube connects the dressing to a suction pump and drainage collection system [11]. VSDs help to effectively accelerate wound healing when used to treat soft tissue injuries [12].

The effectiveness of using VSD to treat open wounds can be enhanced by adding an antimicrobial component to the negative pressure-generating catheter. Adding another catheter with continuous antibiotic irrigation can improve therapy efficacy and lead to a quicker recovery [12]. A recent randomized controlled trial (RCT) showed that VSD is more effective than traditional drainage techniques for treating calcaneal fractures [5].

The study’s conclusion also clarifies the patients’ age distribution and length of recovery. It shows that younger age groups heal wounds more quickly [8]. Age is a determinant in recovery, as previous research has proven. The location, size, and kind of open fractures are among the other variables that raise a patient’s risk of infection and amputation [6].

The emergence of several technical advancements, including irrigation systems, foam dressings with different densities and pore sizes, and silicone interfaces, has helped negative pressure wound therapy (NPWT). Greater flexibility for each unique circumstance is the outcome. However, NPWT is not always suitable and cannot replace an essential surgical treatment. This study aims to review NPWT’s guiding principles, valuable techniques, and hints [11].

In light of these findings, our study used VSD as a primary treatment. Suction dressing has drastically improved the patients’ wounds and prevented severe complications, including leg amputation. Our study plays a vital role in providing an excellent treatment through suction dressing in contrast to the burden of operative definitive management for open calcaneal fractures. It is surgeon-friendly and cost-effective while removing infection and promoting healthy granulation tissue.

Using VSD in managing open calcaneal fractures has demonstrated promising outcomes in our case series. VSD is a valuable adjunct to wound healing and infection control, leading to favorable clinical outcomes and mitigating the risk of serious complications, such as amputation. This approach offers a non-operative, cost-effective alternative that may be particularly beneficial in cases where definitive fracture stabilization is challenging or contraindicated. Further research and larger-scale studies are warranted to validate the efficacy of VSD in managing open calcaneal fractures and establish standardized treatment protocols for optimal patient outcomes.

## Data Availability

Data is available from authors upon reasonable request.
